# Supplementary material for: Predicting visual function by interpreting a neuronal wiring diagram
Source: Nature. 2024 Oct 2;634(8032):113–23. doi: 10.1038/s41586-024-07953-5 (PMC11446822; doi:10.1038/s41586-024-07953-5)
Supplement: Supplementary file 6 — CRF and ERF predictions for individual TmY4 and TmY9 cells. Analogous to Supplementary Data 3, but for TmY target types. Shown are the top four monosynaptic pathways, the strongest pathway passing through each of the top ten intermediary types (ranking from Extended Data Fig. 7), and the trisynaptic pathway Tm1–TmY–Dm3–TmY (see the section entitled Prediction of spatial normalization). [file 41586_2024_7953_MOESM6_ESM.zip › DataS4/TmY9q/720575940623443529.pdf]

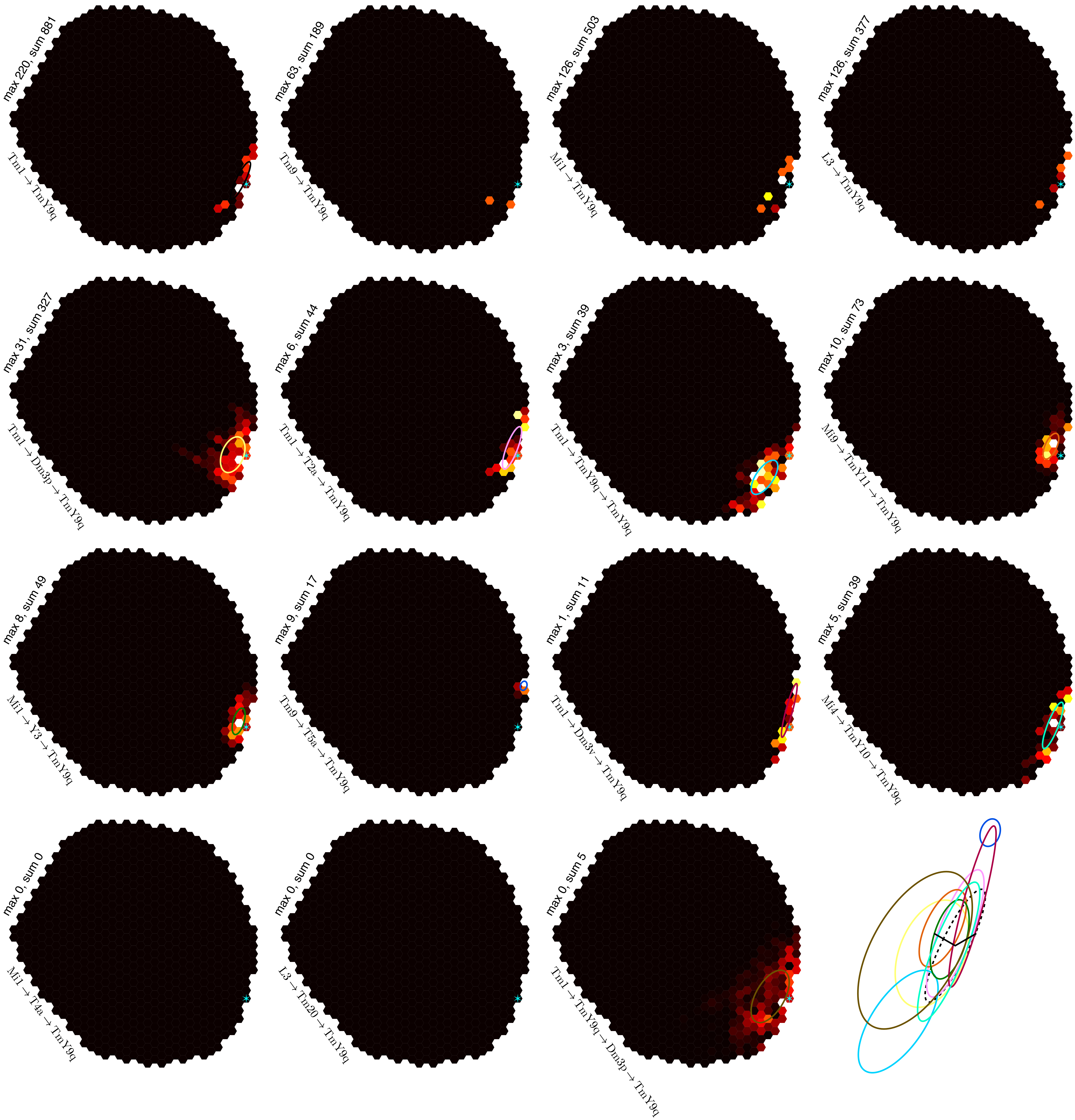
max 220, sum 881
Tm1 → TmY9q
max 63, sum 189
Tm9 → TmY9q
max 126, sum 503
Mi1 → TmY9q
max 126, sum 377
L3 → TmY9q
max 31, sum 327
Tm1 → Dm3p → TmY9q
max 6, sum 44
Tm1 → T2a → TmY9q
max 3, sum 39
Tm1 → TmY9q → TmY9q
max 10, sum 73
Mi9 → TmY11 → TmY9q
max 8, sum 49
Mi1 → Y3 → TmY9q
max 9, sum 17
Tm9 → T5a → TmY9q
max 1, sum 11
Tm1 → Dm3v → TmY9q
max 5, sum 39
Mi4 → TmY10 → TmY9q
max 0, sum 0
Mi1 → T4a → TmY9q
max 0, sum 0
L3 → Tm20 → TmY9q
max 0, sum 5
Tm1 → TmY9q → Dm3p → TmY9q
